# Associations between different types of physical activity and teachers’ perceived mental, physical, and work-related health

**DOI:** 10.1186/1471-2458-14-534

**Published:** 2014-05-30

**Authors:** Inge Bogaert, Kristine De Martelaer, Benedicte Deforche, Peter Clarys, Evert Zinzen

**Affiliations:** 1 Department of Movement Education and Sportstraining, Faculty of Physical Education and Physiotherapy, Vrije Universiteit Brussel, Pleinlaan 2, B-1050 Brussels, Belgium; 2 Department of Human Biometry and Biomechanics, Faculty of Physical Education and Physiotherapy, Vrije Universiteit Brussel, Pleinlaan 2, B-1050 Brussels, Belgium; 3 Department of Movement and Sports Sciences, Faculty of Medicine and Health Sciences, Ghent University, Watersportlaan 2, B-9000 Gent, Belgium

**Keywords:** Well-being, Teachers, Leisure-time physical activity, Occupational physical activity, Job satisfaction, Occupational stress, Absenteeism

## Abstract

**Background:**

The teaching profession is characterized by high levels of stress and physical complaints, which might be improved through regular participation in physical activity (PA). However, the effect of PA on mental and physical health is not always consistent and depends on the type of PA performed. The aim of this study was to examine the mental, physical, and work-related health of Flemish secondary school teachers and identify the impact on those health variables by demographic and teaching-related factors and various types of PA.

**Methods:**

This study included an online survey conducted across a representative sample of secondary school teachers (n = 1066, average age 40 years; 68 percent female). Level of PA and sitting time were estimated using the International Physical Activity Questionnaire, and perceived mental health and physical health were estimated using the Short Form 36. Work-related factors such as job satisfaction, occupational stress, and absenteeism were also collected. T-tests, ANOVAs, and linear regression analyses were performed.

**Results:**

Flemish secondary school teachers have poorer perceived mental and physical health than a general healthy population. This difference is particularly evident among female teachers, who reported lower perceived health, more occupational stress, and more absent days compared to their male colleagues. Higher participation in leisure-time PA was associated with a more positive perceived health. In contrast, higher levels of occupational PA and sitting time had a negative impact on perceived health. Total amount of PA, total amount of moderate-to-vigorous PA, transportation-related PA, and PA at home were not associated to teachers’ perceived health.

**Conclusion:**

Because secondary school teachers’ levels of perceived health are low, they are an important target group for interventions aiming to improve health. Only leisure-time PA was associated with more positive perceived health. This finding may indicate that teachers performing more exercise during leisure time, or in a more autonomous way, may be more resistant to physical and mental health problems. Future research should verify whether promoting leisure-time PA among teachers has the potential to improve their mental and physical health, and counteract the negative associations between teachers’ health and their occupational PA.

## Background

The teaching profession is characterized by a relatively high level of absenteeism and early retirement [1-3]. This association may be caused by poor general well-being attributed to high levels of stress and poor physical health linked to the teaching job [1,4-7]. Research among teachers has indicated that the mental strain of teaching is mainly related to high workloads and adverse events caused by pupils and parents [7-9]. The mental well-being of teachers was found to be worse among female teachers, and also seemed to deteriorate with age [9,10]. Musculoskeletal problems (MSP) among teachers were most prevalent in the back, neck, and upper limbs [11]. Female gender, longer employment in teaching, prolonged periods of standing, and a head-down posture were identified as significant risk factors for the development of neck pain among school teachers [11-14]. Some studies among teachers also identified body weight and waist-hip ratio as risk factors for MSP and reduced work ability [1,15]. Studies among school teachers concluded that work-related factors such as high levels of perceived stress, high workload, low collegiality, and low job satisfaction were significantly associated with a lower mental and physical well-being [11-13,16-18].

These health- and work-related problems among teachers may be alleviated by regular participation in physical activity (PA) [19-21]. However, no previous studies have examined the association between teachers’ levels of PA and their mental, physical, and work-related health. Review studies at various other worksites found evidence of PA interventions having a positive influence on absenteeism, and more tentatively, on job satisfaction and job stress [22,23]. Among a general adult population, regular participation in PA may prevent stress by creating a better social network, higher stress tolerance, better self-esteem, and more active coping behaviors [21,24]. PA may buffer the effect of stress by influencing the individual’s eating patterns, stabilizing hormone production, and lowering blood pressure [21,25]. Current clinical guidelines [26] also recommend PA as a primary and secondary prevention against lower back problems among adults.

However, the effect of PA on mental and physical health is not always consistent and depends on the type of PA performed [26-28]. In the literature, occupational PA was associated with a higher risk of all-cause mortality [29] and lower back pain in the general adult population [26]. Specifically for teachers in Physical Education classes, Lemoyne et al. [30] found occupational PA to be associated with lower back pain. In contrast, leisure-time PA was associated with better self-rated physical health [28,31] and mental health [32,33], and was found to counteract the association between occupational PA and a higher risk of all-cause mortality [29] among a general adult population. In Flemish adults, participation in sports was found to be the only type of PA significantly associated with lower perceived stress compared to other types of PA, such as active commuting, housekeeping, and other forms of leisure-time PA [24,34]. Other studies reported that cycling to work had a positive influence on health-related quality of life in healthy adults [33,35]. Evidence also suggests that, in addition to meeting PA guidelines, decreasing sitting time may have a positive effect on various health factors [36-38].

To date, the relationship between participation in various types of PA and school teachers’ health has not been explored. The current study attempts to address this gap in research by unraveling this complex relationship, which is necessary for the development of teacher-specific PA interventions aiming to improve teachers’ health. The aim of this study was twofold. Firstly, the study examined the impacts of demographic and occupation-related factors on teachers’ perceived mental, physical, and work-related health. Secondly, the study explored associations between various types of PA performed by the teachers, and their perceived mental, physical, and work-related health.

## Methods

### Participants and procedure

Between November 2010 and February 2011, the current study issued an online survey within a representative sample of Flemish secondary school teachers in Belgium. Although education in Belgium is divided into three communities according to their spoken language (Flemish, French, and German), this study addressed only teachers in Flemish-speaking schools, largely because the study was funded by an institution situated in the Flemish community. Moreover, schools in Flanders are categorized according to education network and education form [39]. Three types of education network can be distinguished depending on the schools’ philosophical backgrounds and educational approaches: governmental, free (mainly catholic and method schools), and provincial education. Education forms are categorized as general education on the one hand, and practice-oriented (vocational) education on the other, with technical education in-between.

In total, 105 randomly selected Flemish schools participated in the study (response rate 64 percent), stratified by education network, education form, and province. In each of the schools, the principal was asked to distribute the link to the online survey among the school’s teachers. The research team send three reminder notices to the schools—two weeks, four weeks, and eight weeks after the link was distributed – encouraging the schools to distribute the link again. Due to privacy issues, the researchers were unable to contact the teachers directly and did not know exactly how many teachers received the link to the online survey. Hence, the precise response rate could not be calculated.

The final sample consisted of 1066 participants. The only exclusion criterion was pregnancy. Based on data obtained from the Flemish Ministry of Education [40], our sample was broadly representative of the teaching population in Flanders in terms of education form and education network. In terms of gender, our sample consisted of 10 percent fewer male teachers than expected. The study was approved by the ethical committee of the Vrije Universiteit Brussel.

### Measures

The online survey was developed in order to obtain information on PA, sitting time, perceived mental and physical health, and work-related health. In addition to demographic data (gender, age, BMI), the teachers were asked to provide details on the grade, teaching subject, education form, and education network in which they teach, as well as their total years of employment in education.

### Physical activity and sitting time

The long-form International Physical Activity Questionnaire (IPAQ) was used to estimate each teacher’s amount of PA and sitting time in the past week. The IPAQ was selected because it had served as a valid and reliable instrument in several international studies [41,42]. It consists of the following five categories: PA at work or occupational PA; transport-related PA; PA at home, including domestic and gardening activities; PA during leisure time; and sitting time during transport, general weekdays, and weekends. The intensity and the duration of each type of physical activity was obtained, making it possible to estimate the amount of moderate and high-intensity PA performed during the past week [42]. The internal consistency of the IPAQ questions ranged between 0.42 and 0.82 [43].

### Physical and mental health

The research team measured perceived health status using the Short-form 36 Health Survey (SF-36), which has been proven to be a simple and valid instrument for measuring the generic health status of clinical and non-clinical populations [44]. The instrument contains 36 items that result in eight domains. The eight domains were summarized into two scores: perceived physical health and perceived mental health. Physical health includes the following domains: physical functioning, role limitations due to physical problems, bodily pain, and general perception of health. Mental health includes the domains of energy and vitality, social functioning, mental health, and role limitations due to emotional problems. Each summary score was calculated on a scale from 0–100, with the lowest score indicating a very bad health state and the highest score indicating the best possible health state. The summary scores showed high internal consistency with Cronbach’s α of 0.74 for physical health and 0.83 for mental health [43].

### Work-related health

The three variables used to express work-related health in this study were job satisfaction, occupational stress, and absenteeism. Job satisfaction and occupational stress were measured using the Psychosocial Aspects at Work (PAW) questionnaire. This instrument contains 15 items, which were evaluated on a five-point Likert scale to measure attitudes towards specific aspects at work [45]. Higher values correspond to a higher score on the cluster. A higher score for job satisfaction contributes to better work-related health, while a higher score for occupational stress is negative. The results found a Cronbach’s α of 0.93 for job satisfaction (seven items) and 0.70 for occupational stress (four items) [43]. Absenteeism data was obtained by asking the teachers how many days they had been absent during the past 12 months. The lower the number of absent days, the better – and the higher this variable was interpreted.

### Data analysis

The research team conducted all statistical analyses using SPSS 20.0. One-Sample t-tests were used to compare teachers’ health with a reference value. Impacts of demographic and teaching-related factors on teachers’ mental, physical, and work-related health were explored using t-tests and ANOVAs. Equal variances were tested using the Levene’s test. When two groups did not meet the assumption of equal variances, the Welch-Satterthwaite method was used to correct for this violation.

Correlations between teachers’ health and work-related outcomes were calculated using Pearson coefficients. Associations between PA variables and teachers’ mental, physical, and work-related health were assessed using a linear regression analysis, adjusted for age, BMI, and gender. However, the research team found sitting time and all PA variables to be skewed, so logarithmic transformations were used to improve normality. For all analyses, the significance was set at p < 0.05, and a trend towards significance was noted when p < 0.1.

## Results

### Demographic characteristics

In our respondent group (n = 1066), a majority were women (68 percent) and the mean age was 40.3 ± 9.7 years old, ranging from 21 to 61 years. The average body mass index (BMI) was 24.5 ± 4.1 kg/m^2^, ranging from 17 kg/m^2^ to 44.5 kg/m^2^. Sixty percent of the teachers had a normal BMI, between 18.5 and 25 kg/m^2^. One quarter (25 percent) of the respondent group taught practical courses (Table 1). Within this group, physical education (PE) teachers represented the largest proportion (22 percent), followed by caregiving (15 percent) and home economics (11 percent) teachers. With respect to theoretical courses, the most prevalent subject matter specialties were mathematics (15 percent), Dutch language (13 percent), and French language (12 percent).

**Table 1:** Physical, mental, and work-related health outcomes according to demographic and teaching-related factors and active commuting

	**Total (n = 1066)**	**Mental health**	**Physical health**	**Occupational stress**	**Job satisfaction**	**Absenteeism**
	**%**	**scale 0–100**	**scale 0–100**	**scale 0–20**	**scale 0–35**	**(days per year)**
**Mean (SD)**		74.9(19.3)	76.0(18.6)	16.1(2.6)	27.4(5.8)	7.2(23.0)
**Gender**						
Female	68%	73.7(19.7)	74.9(19.4)	16.3(2.6)	27.8(5.7)	8.33(24.9)
Male	32%	77.7(17.6)*	78.3(17.1)*	15.8(2.4)*	27.0(5.6)	4.7(17.5)*
**Age**						
< 30 years	20%	75.8(18.4)	**80.0(15.5)**^**T (a,b)**^	16.4(2.2)	**28.3(5.0)**^**T (a,c)**^	6.0(19.7)
30–55 years	73%	75.0(19.2)	75.8(19.2)	16.1(2.6)	27.5(5.7)	6.5(20.8)
> 55 years	7%	77.8(17.6)	76.6(17.0)	15.6(2.8)	26.2(6.4)	4.0(10.2)
**BMI**						
< 25 kg/m^2^	56%	74.6(19.2)	**76.9(19.0)**^**T**^	16.2(2.6)	27.4(5.9)	6.9(22.9)
> 25 kg/m^2^	44%	75.6(18.6)	74.3(18.7)	16.2(2.4)	27.7(5.5)	8.4(25.4)
**Experience as a teacher**						
< 5 years experience	19%	73.6(19.6)	77.4(17.4)	16.28(2.54)	27.85(5.92)	3.9(11.6)*
> 5 years experience	81%	75.4(18.9)	75.7(19.0)	16.14(2.53)	27.36(5.72)	8.1(25.0)
**Active transport to school**						
Yes	41%	76.1(18.4)	77.0(18.4)	16.1(2.5)	27.4(5.6)	2.0(18.3)*
No	59%	74.2(19.7)	75.3(18.9)	16.2(2.5)	27.6(5.8)	8.7(25.6)
**Teaching Subject**						
Theoretical courses	74%	75.0(19.3)	**77.2(17.8)**^**T**^	16.3(2.35)	27.4(5.8)	**6.3(20.4)**^**T**^
Practical courses	26%	76.0(17.9)	74.5(20.1)	**15.9(2.6)**^**T**^	27.7(5.3)	9.5(25.9)
**PE teacher**						
PE teacher	6.0%	83.5(15.0)**	79.7(20.1)	14.7(3.2)**	28.1(6.7)	9.0(22.6)
Other teachers	94.0%	74.8(19.0)	76.2(18.5)	16.3(2.4)	27.4(5.7)	7.2(23.4)

PA: physical activity; PE: physical education; SD: standard deviation; (a,b) indicates that this value is significantly different between group a and group b.

Significant results are bold and a symbol was added; ^T^= p < 0.1; * = p < 0.05; ** = p < 0.01.

For mental health, physical health, and job satisfaction, a higher value is better.

For occupational stress and absenteeism, a higher value is worse.

### Mental and physical health

Table 2 presents teachers’ mental and physical health. The research team compared the teachers’ values for mental and physical health to the data representing a Flemish sample of healthy working adults, measured by De Geus et al. [35]. The results indicated that teachers scored significantly lower than the average healthy working adult on seven domains of the SF-36, and on both summary scores. Female teachers had significantly worse physical and mental health than their male colleagues (p < 0.05) (Table 1). The research team also found a significant difference in physical health between younger teachers and middle-aged teachers, with younger teachers having better physical health. Teachers with a BMI less than 25 kg/m^2^ tended to perceive themselves as having better physical health than other teachers (p < 0.1). The research team found no significant impact of teaching experience and mode of transport to school on teachers’ health scores. Teachers who taught theoretical courses tended to report better physical health than those teaching more practical courses (p < 0.1). PE teachers reported significantly better mental health than all other teachers (p < 0.01), but did not have better perceived physical health.

**Table 2:** Health related quality of life scores (Mean scores) of Flemish Secondary school teachers (SF 36) compared to reference values of Flemish adults

	**Current study Mean (SD)**	**Reference values (De Geus et al. **[35]**)**	**T values and significance**
	**(n = 963)**	**(n = 92)**	
**Mental health summary score**	74.9 (19.3)	80.77	-9.45**
Vitality	65.4 (18.2)	70.95	-9.48**
Social functioning	81.9 (21.3)	90.10	-12.03**
General mental health	71.8 (16.6)	75.65	-7.20**
Role limitations due to emotional problems	80.4 (34.9)	86.40	-5.48**
**Physical health summary score**	76.0 (18.7)	78.11	-3.51**
Physical functioning	84.7 (18.4)	91.20	-11.41**
Role limitations due to physical health problems	75.3 (37.1)	80.40	-4.40**
Bodily pain	76.8 (21.4)	85.60	-12.97**
General health perception	67.0 (18.2)	55.25	19.96**

Significant results were given a symbol; ^T^ = p < 0.1; *= p < 0.05; **= p < 0.01.

### Work-related health

Female teachers reported significantly higher levels of occupational stress (p < 0.05) than their male colleagues and were significantly more absent (p < 0.05) (Table 1). Job satisfaction was similar between males and females. Teachers under 30 years of age had a tendency to be more satisfied with their jobs than the two older age groups (p < 0.1). BMI, teaching experience, and mode of transport to school were not related to teachers’ levels of occupational stress and job satisfaction. Teachers with less than five years of teaching experience and teachers using active transport to commute to school reported significantly lower numbers of absent days than their colleagues (p < 0.05). Teachers teaching practical courses tended to feel less occupational stress than their colleagues (p < 0.1), but tended to be more absent than teachers who taught theoretical courses (p < 0.05). PE teachers had lower levels of occupational stress than other teachers (p < 0.01), but did not show a different level of job satisfaction or number of absent days.

### Correlations between teachers’ health and worksite outcomes

Teachers’ physical health was strongly related to their mental health (r = 0.57), and moderately related to job satisfaction (r = 0.31) and to absenteeism (r = -0.28) (Table 3). Teachers’ mental health was strongly related to job satisfaction (r = 0.52) and slightly related to absenteeism (r = -0.22).

**Table 3:** Pearson correlations between teachers’ physical, mental, and work-related health

	**Physical health**	**Occupational stress**	**Job satisfaction**	**Absenteeism**
Mental health	0.574**	-0.188**	0.520**	-0.218**
Physical health		-0.085**	0.306**	-0.280**
Occupational stress			0.091**	0.033
Job satisfaction				-0.163**

Significant results were given a symbol; ^T^= p < 0.1; *= p < 0.05; **= p < 0.01.

### Associations between physical activity patterns and teachers’ physical, mental, and work-related health

The linear regression analysis in Table 4 shows the associations between the various types of PA and teachers’ perceived mental, physical, and work-related health, adjusted for age, BMI, and gender. No associations were found between the total amount of PA (total PA and total Moderate to Vigorous PA or MVPA) and health outcomes. However, different associations were found between the various types of PA and the outcome variables. Occupational PA and sitting time were negatively related to perceived mental health (β = -1.88; p < 0.05 & β = -6.12; p < 0.1) and perceived physical health (β = -2.14; p < 0.05 & β = -9.39; p < 0.01). Additionally, a negative association was found between occupational PA and job satisfaction (β = -0.5; p < 0.05), and a positive association between occupational PA and occupational stress (β = 0.24; p < 0.05). Leisure-time PA was positively related to teachers’ mental health (β = 3.31; p < 0.01), physical health (β = 3.64; p < 0.01), and job satisfaction (β = 0.36; p < 0.1), but negatively related to occupational stress (B = -0.31; p < 0.01) and absenteeism (β = -2.29; p < 0.05). No significant associations were found between active transport or PA performed at home and teachers’ physical, mental, or work-related health.

**Table 4:** Linear regression analysis of different types of PA with teachers’ physical, mental, and work-related health outcomes

	**Mental health**			**Physical health**			**Occupational stress**			**Job satisfaction**			**Absenteeism**		
**Independent covariates**															
	**β**	**SE**	**t**	**β**	**SE**	**t**	**β**	**SE**	**t**	**β**	**SE**	**t**	**β**	**SE**	**t**
Gender	4.01	1.45	2.77**	3.42	1.42	2.41*	-0.50	0.19	-2.62**	-0.84	0.43	-1.93^T^	-3.63	1.77	-2.05*
Age (years)	-0.01	0.07	-0.14	-0.26	0.07	-3.76**	-0.01	0.01	-1.22	-0.05	0.02	-2.19*	0.12	0.09	1.35
BMI (kg/m^2^)	0.01	0.17	0.07	-0.36	0.16	-2.25*	0.01	0.02	0.47	0.03	0.05	0.62	0.28	0.20	1.39
**PA & Sitting time** (minutes a week adjusted for age, BMI, and gender)															
	**β**	**SE**	**t**	**β**	**SE**	**t**	**β**	**SE**	**t**	**β**	**SE**	**t**	**β**	**SE**	**t**
Total PA	-0.58	1.9	-0.3	0.84	1.83	0.46	-0.12	0.25	-0.47	-0.96	0.56	-1.72	-2.21	2.3	-0.97
Total MVPA	-0.5	1.26	-0.4	0.81	1.21	0.67	0.23	0.17	1.36	-0.43	0.37	-1.16	-1.54	1.54	-1,00
Occupational PA	-1.88	0.79	-2.39*	-2.14	0.76	-2.82**	0.24	0.11	2.28*	-0.5	0.23	-2.17*	-0.46	0.96	-0.48
PA in leisure time	3.31	0.74	4.48**	3.64	0.71	5.13**	-0.31	0.10	-3.15*	0.36	0.22	1.62^T^	-2.29	0.91	-2.52*
PA at home	0.44	0.89	0.5	1.35	0.86	1.58	-0.01	0.12	-0.09	0.29	0.26	1.1	0.11	1.08	0.1
Active transport	0.47	0.73	0.64	0.48	0.71	0.68	0.02	0.10	0.2	-0.01	0.22	-0.04	-0.16	0.89	-0.18
Sitting time	-6.12	3.22	-1.90^T^	-9.39	3.09	-3.03**	0.61	0.43	1.43	-0.14	0.95	-0.15	6.1	3.91	1.56

PA: physical activity; MVPA: moderate-to-vigorous physical activity.

Significant results were given a symbol; ^T^ = p < 0.1; * = p < 0.05; ** = p < 0.01.

Mental health, physical health, and job satisfaction are positive variables (a higher value is better).

Occupational stress and absenteeism are negative variables (a higher value is worse).

## Discussion

The aim of this study was to explore the perceived mental, physical, and work-related health of Flemish secondary school teachers and identify the impacts of demographic and teaching-related factors and different types of PA on several health-related variables. To our knowledge, this is the first study to analyze associations between different types of PA performed by teachers and their perceived health. Findings of this study may provide information on which types of PA could be promoted among teachers to enhance their mental, physical, and work-related health.

Firstly, the results of this study indicated that secondary school teachers’ perceived mental and physical health was significantly lower compared to a Flemish sample of healthy working adults [35]. Specifically, female teachers reported significantly lower perceived health than their male colleagues, were more absent and perceived a higher amount of occupational stress. This result is in line with the literature suggesting that higher prevalence of health problems among women may be related to a lower physical strength, lower pain threshold, and higher workload combining careers and household tasks [11,14,46]. In addition, teachers with a BMI higher than 25 kg/m^2^ and older than 30 years tended to show a poorer perceived physical health compared to their colleagues. Other studies examining teachers’ health also found that higher BMI and higher waist-hip ratio are related to lower levels of physical fitness and more physical problems [1,11,14]. In these studies, poorer physical health was also associated with a longer duration of employment, which mostly runs parallel with age. Erick and Smith [11] interpreted this association as a cumulative effect of workload on the musculoskeletal system of teachers.

The findings of this study also indicated that teachers who teach practical courses tended to have a poorer perceived physical health and more absent days. However, in the present study, this finding does not seem to be applicable for PE teachers (6 percent of total respondents), who reported better mental health and less occupational stress compared to their colleagues. Erick and Smith also indicated that PE teachers reported a lower prevalence of MSP than other teachers. They argued that PE teachers might be more physically active and perform more leisure time PA compared to other teachers, and that additional leisure-time activity may enhance their overall fitness. Therefore, it may be useful to tailor PA intervention strategies according to teaching subject area and class environment. However, a more detailed analysis of the relationship between teachers’ health, amount of PA, and teaching subject area is needed in order to confirm this finding.

A second observation in this study was the significant association between occupational PA and poorer perceived health among secondary school teachers. This finding could indicate that occupational PA performed by the teachers may aggravate several mental and physical workplace-related problems. Occupational PA has also been associated with negative health among other adult employees. In a review study by Vuori et al. [26], prolonged occupational PA was associated with symptoms of low back pain. Holtermann et al. [29] found that occupational PA was related to higher all-cause mortality among men. In contrast, in a study by Probert et al. [47] among a representative Canadian sample, a higher amount of occupational PA was associated with greater health benefits. These seemingly conflicting conclusions indicate that the association between occupational PA and populations’ health may vary depending on the occupational group and the specific characteristics of the job. Most occupational activities performed by the teachers (with the exception of some teachers in a practical subject area) do not involve any heavy lifting, isometric work, or repetitive work inherent to occupations with a high physical workload [48]. According to Chiu et al. [13], secondary school teachers generally perform many hours of standing, overhead writing, and sitting with a head-down posture when reviewing student work and preparing lesson plans. Because prolonged sitting, static posture, and insufficient back support were positively associated with both neck pain and low-back problems among teachers [14], we can assume that these specific characteristics of teachers’ occupational PA may induce strain and therefore decrease their perceived physical and mental health [7-9,11]. These conclusions point strongly to the need for a greater emphasis on ergonomic solutions for teachers. Combining those ergonomic advices with a simple exercise program to improve basic strength and core stability [49,50] may be useful to increase teachers’ postural control resulting in a better physical health.

Additionally, teachers who reported a higher amount of sitting time indicated lower perceived mental and physical health. The literature also concludes that a longer sitting time has a negative effect on populations’ physical health [37,51] and contributes to poorer mental health and higher incidence of depression [38].

A third observation in our study showed that leisure-time PA was associated with more positive perceived mental, physical, and work-related health among secondary school teachers. A number of other studies in adults have also confirmed that leisure-time PA is associated with more positive self-rated health and better health in general [28,31,32,34]. Holtermann et al. [29] speculated that leisure-time PA can be more beneficial than other types of PA, due to the dynamic use of large muscle groups and the sufficient time between activities to recover from the exercise. Furthermore, Aszatalos et al. [34] suggested that leisure-time PA may induce a greater sense of autonomy and mastery than other types of PA. These feelings support intrinsic motivation, help sustain adherence in physical activity [52], result in higher self-esteem, and indirectly reinforce social coherence and active coping [24].

In the present study, total PA, total MVPA, PA for transport and PA at home were not associated with higher perceived health among Flemish secondary school teachers. This finding is not in accordance with the literature [19,21]. Specifically for active transport, a review by Shepard [53] concluded that physically active commuting to work (walking and or cycling) offers substantial health enhancing benefits among a working-age population. However, Ohta et al. [33] found that only a commute time of 30 minutes or longer was significantly associated with better mental health status among municipal employees. As the average commute time of teachers in our study was only 17.4 minutes per day [43], this may be too short to attain significant mental and physical health benefits. Additionally, our findings did suggest that teachers using active transport to go to school (whether or not combined with motorized transport) were significantly less absent than their colleagues. These seemingly conflicting results could be explored in an intervention study promoting active transport.

Taking into account the low values for perceived mental and physical health, teachers are an important target group for PA interventions aiming to improve employees’ health. Further, given that teachers’ mental, physical, and work-related health are significantly interrelated, we can assume that improving one component of teachers’ health may indirectly improve the other components.

The findings of this study suggest that promoting PA in an autonomy-supportive way may be an effective strategy to enhance teachers’ health. Alongside the physical advantages, a great benefit of leisure-time PA is participants’ ability to choose the activity for themselves. The participants’ ability to select and perform an activity they enjoy is an important part of their intrinsic motivation and indirectly reinforces active coping [24]. According to the Self Determination Theory, autonomy is one of three major components in supporting intrinsic motivation to foster well-being and health [52,54]. Providing teachers with knowledge, understanding, awareness, and motivation necessary to obtain a physically active lifestyle, may be beneficial for their health.

Nevertheless, providing teachers with methods to cope with the stress and strain of the teaching job is not enough in itself. A number of studies have highlighted the importance of structural and organizational adjustments to improve and simplify the work situation of teachers [5,7,8]. A supportive leadership style by senior management using effective communication, formulating feasible targets, and creating a positive atmosphere among colleagues and in the classroom can prevent stress among teachers and students.

A limitation of the present study is that it is cross-sectional in nature, making it less than useful for examining causal relationships. Future research should analyze whether the findings of this study are also valid in a longitudinal study. Further, the results may have been influenced by selection bias – specifically, active and healthy respondents may have been more interested in participating in the survey. Apart from selection bias, studies using self-reported results are characterized with possible reporting bias, due to selective recall and social desirability. The IPAQ is a sensitive questionnaire for these over-reporting problems [42]. Moreover, an objective measurement of absent days among Flemish teachers by the government [3] suggests underreporting in our study. Measurements indicated an average of 14 absent days per year, per worker, compared to an average of seven days in our study. However, the Flemish results were collected across all educational personnel in Flanders, and are not perfectly comparable to data collected across only secondary school teachers.

Lastly, reference data for quality of life among Flemish adults is scarce. Despite the difference in study design and the small sample size, data yielded by this study was compared to data from De Geus et al. [35]. Results should therefore be interpreted with this limitation in mind.

Nevertheless, this study has certain strong points. Firstly, the study is unique in terms of examining the associations between various types of PA and teachers’ perceived health and worksite outcomes. Secondly, the sample size was quite large and representative in terms of location, school network, and education form.

## Conclusion

Flemish secondary school teachers report poorer perceived mental and physical health than a general healthy population. Specifically, female teachers are an important target group for interventions aiming to improve school teachers’ health. Compared to other types of PA, leisure time PA was associated with better perceived mental, physical, and work-related health. Occupational PA and high amounts of sitting time were related to poorer perceived health. Future longitudinal and experimental studies should examine whether teachers performing more leisure time PA can build up a greater resistance to stress and musculoskeletal problems inherent to their job, and thereby counteract the negative associations between occupational PA and teachers’ health.

## Competing interests

The authors declare that they have no competing interests.

## Authors’ contributions

IB, KDM, EZ, PC, and BD developed the study design. IB collected the data and performed the initial data analysis. KDM, EZ, PC, and BD advised when processing the data. IB drafted the manuscript and all other authors critically reviewed and revised versions of the manuscript. All authors read and approved the final manuscript.

## Pre-publication history

The pre-publication history for this paper can be accessed here:

http://www.biomedcentral.com/1471-2458/14/534/prepub
